# Akutes Engwinkelglaukom und Effusionssyndrom nach Phakoemulsifikation

**DOI:** 10.1007/s00347-020-01202-6

**Published:** 2020-08-15

**Authors:** M. Anwar, T. Brockmann, M. Walckling, T. A. Fuchsluger

**Affiliations:** Klinik und Poliklinik für Augenheilkunde, Universitätsmedizin Rostock, Doberaner Str. 140, 18057 Rostock, Deutschland

**Keywords:** Acetazolamid, Uveales Effusionssyndrom, Sulfonamid-Allergie, Kataraktoperation, Unerwünschte Arzneimittelwirkung (UAW), Acetazolamide, Uveal effusion syndrome, Sulfonamide allergy, Cataract surgery, Adverse drug reaction

## Abstract

Eine 72-jährige Patientin entwickelte nach unkomplizierter Kataraktoperation ein beidseitiges sekundäres iridokorneales Engwinkelglaukom bei uvealem Effusionssyndrom. Als Ursache für die Entstehung des Effusionssyndroms konnte die postoperative Einnahme von Acetazolamid ausgemacht werden. Unter Berücksichtigung einer Sulfonamid-freien systemischen und lokalen drucksenkenden sowie antiinflammatorischen Therapie zeigte sich eine schnelle Befundbesserung. Der Fall verdeutlicht eine seltene, aber klinisch schwerwiegende unerwünschte Arzneimittelwirkung von Acetazolamid und zeigt effiziente Therapiemöglichkeiten auf.

## Abstract (en)

A 72-year-old female patient developed bilateral secondary iridocorneal angle-closure glaucoma with uveal effusion syndrome after uncomplicated cataract surgery. The postoperative intake of acetazolamide was identified as causative for the development of the effusion syndrome. Taking a sulfonamide-free systemic and local intraocular pressure lowering and anti-inflammatory treatment into account, a rapid improvement of the ocular manifestation was achieved. The case illustrates a rare but clinically severe adverse effect of acetazolamide and outlines efficient treatment options.

## Anamnese

Eine 72-jährige Patientin stellte sich in unserer Klinik als Notfall mit starken Kopfschmerzen, Übelkeit und Erbrechen sowie beidseitig plötzlicher Sehverschlechterung vor. Am späten Vormittag des Tages erhielt sie an ihrem rechten Auge eine elektive ambulante Kataraktoperation unter Lokalanästhesie. Die Kataraktoperation des Partnerauges erfolgte bereits 2 Wochen zuvor. An beiden Augen verliefen die Operationen komplikationslos mit Implantation einer Intraokularlinse in den intakten Kapselsack. Postoperativ wurde der Patientin, neben der antibiotischen und antiinflammatorischen Lokaltherapie eine prophylaktische orale Einmaldosis Acetazolamid 250 mg verordnet, um einem möglichen postoperativen intraokularen Druckanstieg vorzubeugen. Die Patientin nahm die Acetazolamid-Tablette 4 h nach der Operation ein und legte sich schlafen. Etwa 6 h später wachte sie aufgrund starker Kopfschmerzen auf und bemerkte beidseits eine massive Sehverschlechterung, woraufhin sie die Klinik aufsuchte.

## Klinischer Befund

**Figure 1:**
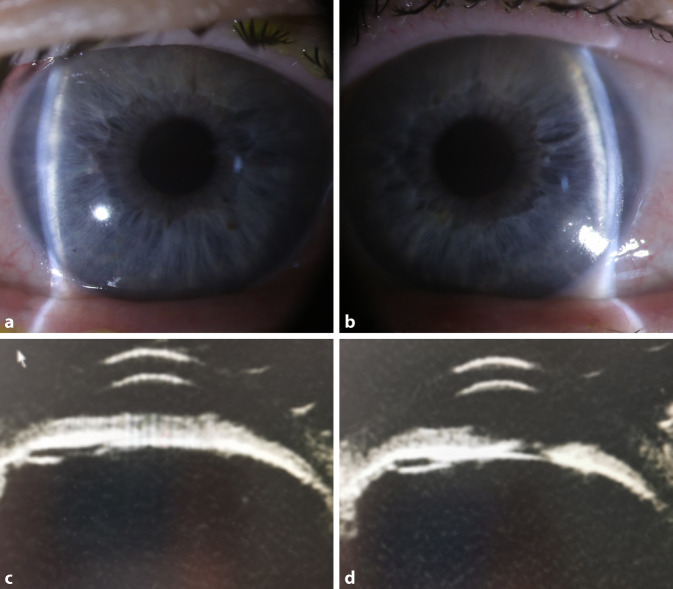

**Figure 2:**
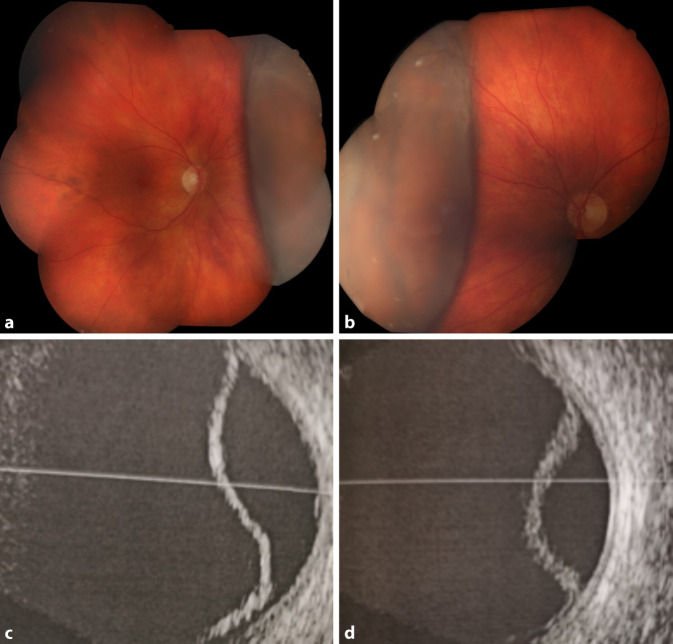

Bei der Erstvorstellung betrug die bestkorrigierte Sehschärfe der Patientin rechts 1/35 und links 1/25 bei einer Refraktionskorrektur rechts von −3,0 dpt und links −3,5 dpt. Spaltlampenmikroskopisch zeigten sich beidseits eine Bindehautinjektion, ausgeprägtes Hornhautödem sowie eine flache Vorderkammer mit Anteflexion der Intraokularlinse (Abb. 1a, b). Zur weiteren Diagnostik führten wir eine Ultraschallbiomikroskopie durch (Abb. 1c, d). Hierbei betrug die Vorderkammertiefe rechts 2,08 mm und links 2,74 mm, der Kammerwinkel zeigte sich bei einem iridokornealen Winkelblock zirkulär verschlossen. Der Intraokulardruck betrug initial rechts 62 mm Hg und links 64 mm Hg. Eine Fundoskopie war aufgrund des ausgeprägten Hornhautödems nicht möglich. In der B‑Scan-Sonographie zeigte sich beidseits eine Aderhautschwellung im Sinne einer Aderhautabhebung (Abb. 2c, d). Die Patientin wurde mit der Arbeitsdiagnose sekundäres Engwinkelglaukom mit Pupillarblock stationär aufgenommen.

## Therapie und Verlauf

Eine initiale systemisch-intravenöse Drucksenkung erfolgte mit Acetazolamid 500 mg und Mannitol (15 %) 300 ml sowie beidseits lokal mit Timolol 0,5 %, Apraclonidin 5 % und Prednisolon 1 %. Unter der genannten Therapie zeigte sich nur eine unzureichende Drucksenkung bei Zunahme der Aderhautschwellung, sodass die Arbeitsdiagnose überdacht werden musste. Acetazolamid wurde im Weiteren als Ursache für ein bilaterales uveales Effusionssyndrom mit sekundärem iridokornealem Engwinkelglaukom angenommen und daher abgesetzt. Dementsprechend erfolgte die weitere systemische Therapie mit einer erneuten Einmalgabe von Mannitol (15 %) 300 ml intravenös und Prednisolon 100 mg per os täglich. Hierunter verbesserte sich der klinische Befund über 3 Tage allmählich. Der Augeninnendruck konnte rechts auf 7 mm Hg und links 9 mm Hg gesenkt werden. In der Fundoskopie zeigte sich noch eine bilaterale periphere Aderhautabhebung (Abb. 2a, b). Der bestkorrigierte Dezimalvisus stieg auf rechts 0,8 und links 0,9 an, die Konfiguration der Vorderkammer normalisierte sich, und die Myopisierung zeigte sich rückläufig. Die systemische und lokal drucksenkende Therapie wurde beendet.

**Figure 3:**
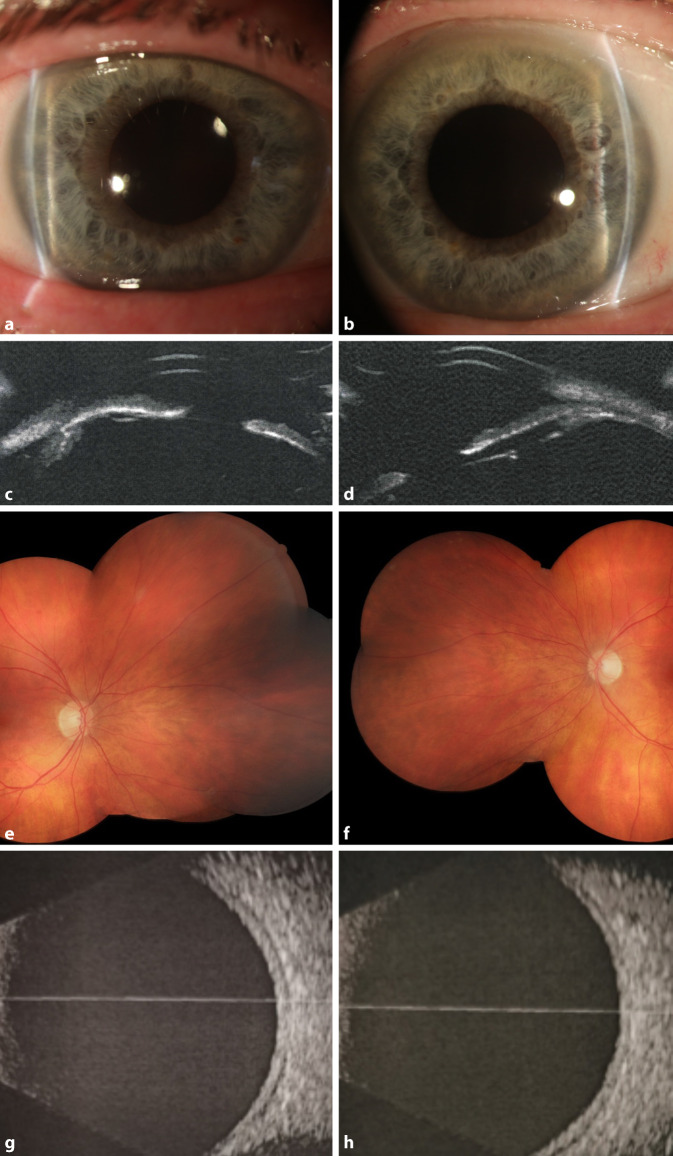

In der Verlaufskontrolle zeigte sich nach 1 Woche beidseits ein regelrechter sonographischer und fundoskopischer Befund des hinteren Augenabschnitts (Abb. 3). Die Vorderkammertiefe betrug rechts 4,39 mm und links 4,37 mm bei einem Augeninnendruck von rechts 10 mm Hg und links 11 mm Hg. Der unkorrigierte Dezimalvisus betrug nun beidseits 1,0. In der nochmals durchgeführten Ultraschallbiomikroskopie offenbarte sich nun beidseits eine Plateau-Iris-Konfiguration (Abb. 3c, d).

## Diagnose

Beidseitiges akutes Engwinkelglaukom bei Acetazolamid-indiziertem Effusionssyndrome nach Kataraktoperation in Plateau-Iris-Konfiguration.

## Diskussion

Die Entwicklung eines uvealen Effusionssyndroms ist bei Sulfonamid-haltigen Arzneimitteln, zu denen auch der Carboanhydrasehemmer Acetazolamid gehört, grundsätzlich bekannt [9]. Der vorliegende Fall verdeutlicht allerdings, dass die hierbei eintretende Aderhautschwellung nicht nur Aderhautfalten hervorruft, sondern foudroyant verlaufen und dabei durch eine massive intraokulare Volumenverschiebung zu einem iridokornealen Winkelblock führen kann. Neben Acetazolamid sind aus der Literatur ähnliche Fälle auch durch andere Sulfonamid-haltige Medikamente wie dem als Antiepileptikum verwendeten Carboanhydrasehemmer Topiramat und dem Thiaziddiuretikum Hydrochlorothiazid bekannt [4, 7, 8]. In der Allgemeinbevölkerung wird die Inzidenz von Sulfonamid-Allergien mit 3–8 % angenommen [2]. Die meisten Reaktionen auf Sulfonamide resultieren aus multifaktoriellen immunologischen und toxischen Stoffwechselmechanismen, während über die genauen Reaktionsmechanismen weniger bekannt ist [1]. Immunglobulin E(IgE)-vermittelte Typ-1-Immunreaktionen auf Sulfonamide können Anaphylaxie, Angioödeme und Urtikaria hervorrufen und nehmen darüber auch Einfluss auf die Konstitution der Aderhaut [10]. Über diesen Weg kann u. a. Acetazolamid, wie in dieser Kasuistik geschildert, paradoxe Nebenwirkungen mit ausgeprägtem uvealem Effusionssyndrom hervorrufen. Interessanterweise wurde festgestellt, dass gerade bei IgE-vermittelten Typ-1-Immunreaktionen nicht die Sulfonamid-definierende NH_2_-SO_2_-Struktur, sondern der im Molekül enthaltene heterozyklische N‑Ring an IgE bindet [3]. Somit ist auch zu erklären, dass verschiedene Sulfonamid-haltige Arzneimittel mit unterschiedlichen heterozyklischen N‑Ringen sehr variable Immunreaktionen hervorrufen können. In dem vorliegenden Fall war bei der Patientin keine Sulfonamid-Allergie bekannt. Dennoch sollte gerade vor der Verabreichung von Acetazolamid und anderen Sulfonamiden eine allergologische Anamnese erfolgen. Gleichzeitig sollte auch bei Vorliegen einer klaren klinischen Situation wie der eines Winkelblocks durch eine spezifische Anamnese differenzialdiagnostisch an die Unterscheidung zwischen einem primären und sekundären Engwinkelglaukom gedacht werden, da dies für die Therapieentscheidung – wie in diesem Fall – von wesentlicher klinischer Relevanz sein kann. In diesem Kontext wurde in der Literatur das Vorliegen einer Plateau-Iris-Konfiguration als wesentlicher Risikofaktor für die Entwicklung eines Winkelblockglaukoms diskutiert [5]. Während lokale und/oder systemische Carboanhydrasehemmer im Rahmen von Augeninnendruckentgleisungen als First-line-Therapeutika angesehen und verwendet werden können [6], wären diese in dem vorliegenden Fall kontraindiziert. Nicht zuletzt sollte neben der adäquaten Therapie auch daran gedacht werden, den Patienten über das Vorliegen und die Bedeutung einer Sulfonamid-Allergie aufzuklären.

## Fazit für die Praxis

- Ein beidseitiges sekundäres iridokorneales Engwinkelglaukom auf Grundlage eines uvealen Effusionssyndroms kann als seltene, klinisch schwerwiegende unerwünschte Arzneimittelwirkung von Acetazolamid eintreten.
- Die Verordnung von Acetazolamid sollte mit Bedacht erfolgen, und mögliche Sulfonamid-Allergien müssen berücksichtigt werden.
- Eine effiziente und sichere Möglichkeit der Akutbehandlung besteht in der Verwendung von Osmodiuretika und Kortikosteroiden.

## References

[1] Dibbern DA, Montanaro A (2008). Allergies to sulfonamide antibiotics and sulfur-containing drugs. Ann Allergy Asthma Immunol.

[2] Giles A, Foushee J, Lantz E (2019). Sulfonamide Allergies. Pharmacy (Basel).

[3] Harle DG, Baldo BA, Wells JV (1988). Drugs as allergens: detection and combining site specificities of IgE antibodies to sulfamethoxazole. Mol Immunol.

[4] Malagola R, Arrico L, Giannotti R (2013). Acetazolamide-induced cilio-choroidal effusion after cataract surgery: unusual posterior involvement. Drug Des Devel Ther.

[5] Man X, Costa R, Ayres BM (2016). Acetazolamide-induced bilateral ciliochoroidal effusion syndrome in plateau iris configuration. Am J Ophthalmol.

[6] Mincione F, Scozzafava A, Supuran CT (2008). The development of topically acting carbonic anhydrase inhibitors as antiglaucoma agents. Curr Pharm Des.

[7] Parthasarathi S, Myint K, Singh G (2007). Bilateral acetazolamide-induced choroidal effusion following cataract surgery. Eye (Lond).

[8] Roh YR, Woo SJ, Park KH (2011). Acute-onset bilateral myopia and ciliochoroidal effusion induced by hydrochlorothiazide. Korean J Ophthalmol.

[9] Senthil S, Garudadri C, Rao HB (2010). Bilateral simultaneous acute angle closure caused by sulphonamide derivatives: a case series. Indian J Ophthalmol.

[10] Wulf NR, Matuszewski KA (2013). Sulfonamide cross-reactivity: is there evidence to support broad cross-allergenicity?. Am J Health Syst Pharm.

